# Screening and characterization of hypothetical proteins of *Plasmodium falciparum* as novel vaccine candidates in the fight against malaria using reverse vaccinology

**DOI:** 10.1186/s43141-021-00199-y

**Published:** 2021-07-16

**Authors:** Claire Aguttu, Brenda Apio Okech, Ambrose Mukisa, George William Lubega

**Affiliations:** 1 Department of Biochemistry and Sports Science, College of Natural Sciences, Makerere University, P.O. Box 7062, Kampala, Uganda; 2 Uganda Virus Reseach Institute, P.O. Box 49, Entebbe, Uganda; 3 Department of Bio-molecular Resources and Bio-lab Sciences, School of Biosecurity, Biotechnology and Laboratory Sciences (SBLS), College of Veterinary Medicine, Animal Resources and Biosecurity, Makerere University, P.O Box 7062, Kampala, Uganda

**Keywords:** Malaria, Epitopes, *Plasmodium falciparum*, Vaccine, Reverse vaccinology, Hypothetical protein

## Abstract

**Background:**

*Plasmodium falciparum* is the most deadly and leading cause of morbidity and mortality in Africa. About 90% of all malaria deaths in the world today occur in Sub-Saharan Africa especially in children aged < 5 years. In 2018, it was reported that there were 228 million malaria cases that resulted in 405,000 deaths from 91 countries. Currently, a fully effective and long-lasting preventive malaria vaccine is still elusive therefore more effort is needed to identify better effective vaccine candidates. The aim of this study was to identify and characterize hypothetical proteins as vaccine candidates derived from *Plasmodium falciparum* 3D7 genome by reverse vaccinology.

**Results:**

Of the 23 selected hypothetical proteins, 5 were predicted on the extracellular localization by WoLFPSORTv.2.0 program and all the 5 had less than 2 transmembrane regions that were predicted by TMHMMv2.0 and HMMTOP programs at default settings. Two out of the five proteins lacked secretory signal peptides as predicted by SignalP program. Among the 5 extracellular proteins, 3 were predicted to be antigenic by VaxiJen (score ≥ 0.5) and had negative GRAVY values ranging from − 1.156 to − 0.440. B cell epitope prediction by ABCpred and BCpred programs revealed a total of 15 antigenic epitopes. A total of 13 cytotoxic T cells were predicted from the 3 proteins using CTLPred online server. Only 2 out of the 13 CTL were antigenic, immunogenic, non-allergenic, and non-toxic using VaxiJen, IEDB, AllergenFp, and Toxinpred servers respectively in that order. Five HTL peptides from XP_001351030.1 protein are predicted inducers of all the three cytokines. STRING protein–protein network analysis of HPs revealed XP_001350955.1 closely interacts with nucleoside diphosphate kinase (PF13-0349) at 0.704, XP_001351030.1 interacts with male development protein1 (Mdv-1) at 0.645, and XP_001351047.1 with an uncharacterized protein (MAL8P1.53) at 0.400.

**Conclusion:**

Reverse vaccinology is a promising strategy for the screening and identification of antigenic antigens with potential capacity to elicit cellular and humoral immune responses against *P*. *falciparum* infection. In this study, potential vaccine candidates of *Plasmodium falciparum* were identified and screened using standard bioinformatics tools. The vaccine candidates contained antigenic and immunogenic epitopes which could be considered for novel and effective vaccine targets. However, we strongly recommend in vivo and in vitro experiments to validate their immunogenicity and protective efficacy to completely decipher the vaccine targets against malaria.

## Background

Malaria is caused by protozoan parasites of the genus Plasmodium: *Plasmodium falciparum, Plasmodium vivax, Plasmodium ovale, Plasmodium malariae*, and *Plasmodium knowlesi* transmitted to people through a bite of an infected female Anopheles mosquito vector. However, *Plasmodium falciparum* is the most deadly and leading cause of morbidity and mortality predominantly in Africa. About 90% of all *malaria* deaths in the world today occur in *Sub-*Saharan Africa especially in children aged < 5 years [1]. In 2018, it was reported that there were 228 million malaria cases that resulted in 405,000 deaths from 91 countries [2]. Some of the malaria symptoms may include body weakness, headache, fever, and shivers [3]. In case of misdiagnosis coupled with delayed treatment, the patient may develop anemia, kidney failure, cerebral malaria, retinopathy, and convulsions. Malaria is commonly managed through the use of antimalarial drugs mainly artemisinin-based combination therapy and indoor residual spraying. Unfortunately, the drug and vector control intervention are being threatened by the ever emerging antimalarial drug and insecticidal resistance which has resulted in an increase of malaria transmission worldwide [4].To-date, there is no efficacious vaccine available globally so far against malaria. Currently, a number of vaccines for malaria are in both pre-clinical and clinical development, targeting both children and pregnant women [5]. These are categorized as pre-erythrocytic vaccines, blood-stage vaccines, transmission-blocking vaccines, and combination vaccines targeting the different stages of the malaria parasite’s life-cycle. Some of the prime candidates include the merozoite surface antigens like merozoite surface protein-1 and apical membrane antigen-1 which have shown moderate effects against the malaria parasite [6, 7]. Malarial vaccine development is hampered by factors such as multiple stages of the life-cycle, multiple antigens per stage, multiple epitopes per antigen, multiple arms of the immune system, multiple immune responses in different hosts, and multiple strains of the parasite [8]. RTS,S is the most advanced malaria vaccine candidate and is based on a virus-like particle containing central repeat and C-terminal epitopes of the major sporozoite surface antigen, circumsporozoite protein. However, it has limitation of waning vaccine efficacy over time with a significant reduction by 3 years post-immunization [9]. Another noticeable limitation of the RTS,S vaccine is the incapability to induce CD8+ T cell responses, which represent an efficient anti-parasite mechanism that eliminates malaria liver stages (reviewed in [10]. It is therefore acceptable that identifying new targets which may be more efficacious is paramount. Initially, the *P. falciparum* 3D7 nuclear genome contained 5300–5400 protein-coding genes and 60% (3208) had unknown functions [11]. However, the number of Plasmodium-predicted genes has since risen to 5438 [12] and approximately 50% have no ascribed function [13, 14] and are also known as hypothetical proteins (HP)**.** Hypothetical proteins are sequences with little to no experimental evidence for their function’s existence being characterized by a low identity to proteins with known function [15]. Two groups of HPs exist: uncharacterized protein families and domains of unknown function. Many studies have identified and characterized hypothetical proteins from different microorganisms which appear to be of great importance [16–21]. Reverse vaccinology (RV) is a new approach to identify drug target and vaccine candidates without the need for culturing the parasite [22]. Through the use of online bioinformatics algorithms, potential peptide-based vaccine antigens notably the serogroup B Neisseria meningitides vaccine and later staphylococcus vaccine were identified and developed successful [23, 24]. Reverse vaccinology analyzes the entire parasites’ protein repertoire using bioinformatics tools to prioritize potential targets for experimental validation both either in in vitro or in vivo*.* Thus, identifying new target antigens is the another way of boosting up new malaria vaccine development [25]. Moreover, for the probable antigens to be potentially good vaccine candidates, they must be surface exposed and able to be recognized by the host’s immune system [26]. This study was designed to employ RV and immunoinformatics approaches to identify potential vaccine targets with their epitopes that can produce the B and T cell-mediated immunity. These predicted epitopes could be considered as promising candidates for effective peptide-based vaccine against malaria.

## Methods

### Protein selection and retrieval

Hypothetical proteins were searched in National Center for Biotechnology information (NCBI) database by typing the keywords “conserved hypothetical proteins *Plasmodium falciparum*” and a total of 23 protein sequences (accession nos. XP_002808602.1, XP_001350996.2, XP_002808605.1, XP_001350997.1, XP_001351014.2, XP_001350955.1, XP_001351004.1, XP_001351002.1, XP_001351011.2, XP_001351013.1, XP_024328987.1, XP_001351030.1, XP_001351040.1, XP_002808611.1, XP_001351044.1, XP_001351047.1, XP_001351049.1, XP_001351045.1, XP_001350986.1, XP_001350982.1, XP_002808604.1, XP_001350978.2, XP_002808603.2) of conserved hypothetical proteins of *Plasmodium falciparum* 3D7 were selected for this study. The protein sequences were selected based on the criteria that they had to be conserved and their function was unknown hence hypothetical. The proteins were then characterized by several bioinformatics tools including WoLFPSORT, SignalP, TMHMM, BLASTP, VaxiJen, and ProtParam. For immunoinformatics, BCpred and ABCpred were used for B cell epitope prediction while CTLpred was employed for T cell epitope prediction.

### Subcellular localization of the hypothetical proteins

Predicting the subcellular location is one of the major criteria for designing a vaccine as immune cells do readily recognize surface exposed proteins on a pathogen. Therefore, subcellular locations of the 23 proteins were predicted using WoLFPSORTv2.0 [27] which is a free online server localized at www.wolfpsort.org. WoLFPSORT is an extension of the PSORT II program which converts protein amino acid sequences into numerical localization features, based on sorting signals, amino acid composition, and functional motifs such as DNA-binding motifs. The method groups proteins in more than 10 locations with an estimated sensitivity and specificity of around 70% for nucleus, mitochondria, cytosol, plasma membrane, extracellular, and chloroplast. Only those proteins that were localized on the extracellular site of the pathogen were selected for further analysis.

### Antigenicity analysis

Antigenicity of the 5 extracellular proteins chosen from the previous step was checked using the VaxiJen2.0 online server (http://www.ddg-pharmfac.net/vaxijen/VaxiJen/VaxiJen.html). VaxiJen is an alignment-free approach for antigen prediction with an accuracy of 70 to 89% hence a crucial tool in reverse vaccinology. The method is based on auto cross covariance transformation of protein sequences into uniform vectors of principal amino acid properties. The method threshold value was set to 0.5%. Hence, any protein that had an antigenic score above 0.5% was selected for further analysis [28].Proteins with VaxiJen score less than 0.5% were considered non antigenic and were therefore discarded.

### Prediction of transmembrane helices (TM) and signal peptide

The three antigenic (VaxiJen ≥ 0.5%) hypothetical proteins selected from the previous step were characterized for transmembrane domains using TMHMM based on hidden Markov model (http://www.cbs.dtu.dk/services/TMHMM/) [29] and HMMTOP (http://www.enzim.hu/hmmtop/) [30] at a default setting of the parameters. Proteins having ≤ 1 transmembrane helices by both methods were selected as they are considered to be good targets because of their easy to clone and express during experimental validation studies.

SignalP ver.5.0 server (http://www.cbs.dtu.dk/services/SignalP/) [31] was used to identify the location of signal peptide within the selected proteins. Proteins with predicted signal peptide were analyzed further.

### Identification of non-human homologous proteins

It is important that potential vaccine targets are not human homologs to avoid autoimmune reactions as the immune system targets cells and proteins it considers “non-self” under normal conditions. In this regard, the three proteins chosen from the previous steps were subjected to a blast analysis using NCBI-BLASTp (https://blast.ncbi.nlm.nih.gov/Blast) against the human proteome as described by Altshul and co-workers [32]. The expectation value (E value) which assesses the statistical significance of BLAST was kept at 0.005 and identity at < 35%. Proteins with E value above 0.005 and < 35% identity were considered non-human homologs and are expected not to interfere with normal host immune mechanism when used as vaccine candidates [33, 34]

### Identification of conserved identity with other Plasmodium strains

Proteins screened from the previous steps were assessed for conservation in the different related Plasmodium strains *(Plasmodium vivax, Plasmodium ovale, Plasmodium malariae*, and *Plasmodium yoeli*) using BLASTp analysis on the NCBI server. This analysis serves to identify functionally conserved proteins which are shared by two or more species. The identity percentage and minimum query coverage were set to 80% and 50% respectively. Hence, all proteins with a sharing percentage ≥ 80% were considered as orthologous conserved.

### Allergenicity and antibody production predictions

Allergenicity was checked by two different methods including AllerTOP.v2.0 (http://www.pharmfac.net/allertop) and AllergenFP.v1.0 (http://ddg-pharmfac.net/Allergen FP). AllergenFP.v1.0 uses amino acid E-descriptors and auto- and cross-covariance transformation of protein sequences into uniform equal-length vectors to predict allergens [35]. Proteins not having allergic properties by all the two prediction methods were considered for further analysis. IgPred [36] does predict the potential antibody (Ab) isotype which can be elicited by a particular protein with an accuracy of around 80%. We employed IgPred online server (http://crdd.osdd.net/raghava/igpred/) to predict the different antibody subtypes that might be elicited by the selected hypothetical proteins.

### Physico-chemical parameters analysis

The physicochemical properties, amino acid composition, molecular weight (Mw), theoretical isoelectric point (pI), instability index (II), extinction coefficient (EC), half-life, and grand average of hydropathy (GRAVY) of the non-allergenic proteins were analyzed using ProtParam server (https://web.expasy.org/protparam/) [37]. Instability index predicts protein’s stability in the test tube whereby an II value (< 40) is said to be stable and vice versa. Aliphatic index value explains vaccines thermostability and is defined as the relative volume occupied by the aliphatic side chain amino acids. GRAVY values explain the hydrophilic or hydrophobic nature of the protein and are calculated as the sum of all hydropathy values of all the amino acids divided by the number of residues in the sequence [38].

### Prediction of B and CTL antigenic epitopes

Accurate identification of antigenic epitopes on a protein is important for the development of immunodiagnostic kits, synthetic peptide vaccines, and antibody production [39]. B cell epitopes were predicted on the three selected hypothetical proteins using prediction methods namely ABCpred (http://crdd.osdd.net/raghava/abcpred/) [40] and BCpred software (https://omictools.com/bcpreds-tool).The length of the B cell epitopes was fixed at 16 and the cutoff at 0.51 in ABCpred. For BCpreds predictions, 20 mers peptides were identified at a specificity of 70%. ABCpred uses artificial neural network (ANN) which is a machine learning system inspired by biological neural network to find patterns in a given dataset. BCpreds contains two methods based on different algorithms namely amino acid pair (AAP) antigenicity method and BCpreds method using subsequence kernel [41]. The B cell epitopes resulting from the three algorithms were assembled and the overlapping regions were selected as predicted B cell epitopes. Subsequently, the selected B cell epitopes were screened for their antigenicity, allergenicity, and toxicity using VaxiJen v2.0, AllergenFP v1.0, and ToxinPred server (http://crdd.osdd.net/raghava/toxinpred/) respectively. CTLPred server (http://crdd.osdd.net/raghava/ctlpred/) [42] a direct method for predicting CTL epitopes from an antigenic sequence was used to predict cytotoxic T cell epitopes by a combined approach of artificial neural network (ANN) and support vector machine (SVM) learning technique at a cutoff score of 0.51 and 0.36, respectively, above which peptides are considered to be antigenic. The selected T cell epitopes were analyzed for their antigenicity, immunogenicity, allergenicity, and toxicity using VaxiJen2.0, IEDB (http://tools.iedb.org/immunogenicity/) programs, AllergenFPv 1.0, and Toxinpred servers, respectively.

### Prediction of helper T-lymphocyte (HTL) epitopes

Helper T-lymphocyte (HTL) induces both humoral and cellular immune responses. Hence, HTL epitopes are most likely to play a significant role in preventive and immunotherapeutic vaccines. We applied the IEDB MHC-II binding tool (http://tools.iedb.org/mhcii/) to predict 15 amino acid long HTL epitopes using NN-align method [43]. NN-align method generated a percentile rank by comparing peptide’s binding affinity with a comprehensive set of randomly selected peptides from the Swiss-Prot database. For this study, peptides with a percentile rank ≤ 5 were considered for further analysis [44]. The selected HTL peptides were assessed for antigenicity and cytokine induction particularly interferon-gamma (IFN-γ), interleukin-4 (IL-4), and interleukin-10 (IL-10). For predicting antigenicity, interleukin-4 (IL-4) and interleukin-10 (IL-10), VaxiJen, IL4pred (http://crdd.osdd.net/raghava/il4pred/), and IL10pred (http://crdd.osdd.net/raghava/IL-10pred/) servers, respectively, were used [45, 46]. In order to predict IFN-γ inducing HTL epitopes, we employed IFNepitope server (http://crdd.osdd.net/raghava/ifnepitope/) using a hybrid method (Motif and SVM) along with IFN-gamma versus non-IFN-gamma model [47].

### Protein–protein interaction analysis

This was aimed at understanding the functional pathway and interaction of the hypothetical proteins with closely related proteins. STRINGv10.5 web server (https://string-db.org/) was used to predict this interaction by choosing the query sequences and protein–protein interaction networks were generated [48].

## Results

### Protein sequence retrieval and subcellular localization analysis

**Fig. 1:**
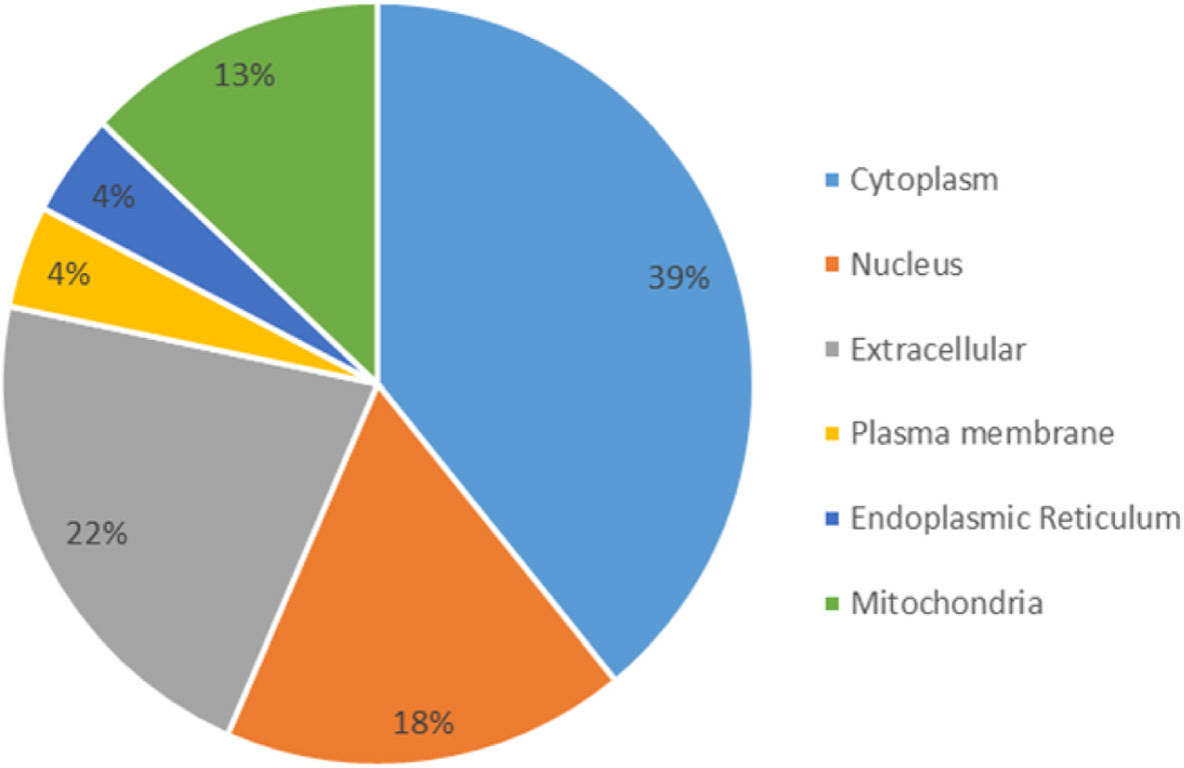
Subcellular localization prediction using WOLFPsort web server of the 23 hypothetical proteins of *P. falciparum*

Twenty three hypothetical proteins of *Plasmodium falciparum* with amino acid length ranging from 81 to 2221 were retrieved from NCBI. These were then submitted to WoLFPSORT web server for subcellular localization. The prediction revealed 9(39%), 4(18%), 5(22%), 1(4%), 1(4%), and 3(13%) are localized in the cytoplasm, nucleus, extracellular, plasma membrane, endoplasmic reticulum, and mitochondria, respectively. The results of subcellular localization analysis are given in Fig. 1.

### Antigenicity analysis

The antigenicity of the 5 extracellular hypothetical proteins was calculated using VaxiJen ver. 2.0. Of these, 3 extracellular proteins were found to have antigenicity score above the threshold value of 0.5 (antigenic). Hypothetical proteins with VaxiJen score above 0.5 are shown in Table 1. Two extracellular hypothetical proteins XP_001351049.1 and XP_001350982.1 were eliminated at this step for having an antigenicity score lower than 0.5 which were considered as non-antigens.

**Table 1:** Characteristics of 3 selected extracellular hypothetical proteins of *Plasmodium falciparum*

No.	NCBI accession number	No. of amino acids	Subcellular localization	TM helices	SignalP	VaxiJen score	Allergenicity	Ig subtype
1	XP_001350955.1	117	Extracellular	0	Yes	0.7376	No	IgG
2	XP_001351030.1	234	Extracellular	1	Yes	0.5191	No	IgG
3	XP_001351047.1	81	Extracellular	0	No	0.8264	No	-

*Tm* Transmembrane, *Ig* immunoglobulin*, IgG* immunoglobulin G

### Prediction of transmembrane domains and Signal peptide

Characteristic of transmembrane helices in proteins was predicted using TMHMM based on hidden Markov model and HMMTOP programs at a default setting of the parameters. As per the predictions, all the 3 antigenic extracellular hypothetical proteins were observed to contain none or 1 transmembrane domain (Table 1). SignalPv5.0 predicted a signal peptide on two proteins (NCBI: XP_001350955.1 and XP_001351030.1) and no signal peptide was found on XP_001351047.1 protein (Table 1). By using AllerTop and AllergenFP webservers to predict allergenic proteins and IgPred to predict the immunoglobulin subtype induced by the proteins, all the three hypothetical proteins were non-allergens. Hypothetical proteins XP_001350955.1 and XP_001351030.1 were predicted to induce IgG while for XP_001351047.1 no immunoglobulin subtype (Table 1).

### Screening for non-human homologs

In order to avoid interference against host immune mechanism, it is critical that potential vaccine candidates are non-human homologous. Consequently, the 3 hypothetical proteins selected from the previous steps were subjected to BLASTp search against human proteome. All the 3 extracellular proteins, namely XP_001350955.1, XP_001351030.1, and XP_001351047.1 had no significant similarity with human proteome (Table 2).

**Table 2:** Homology and conservation characteristics of 3 extracellular hypothetical proteins of *Plasmodium falciparum*

No. NCBI accession No.	Amino acid sequence homology percentage in Plasmodium strains (%)			Human proteome homology
	*P. vivax*	*P. ovale*	*P. yoeli*	
1. XP_001350955.1	-	-	-	No significant similarity found
2.XP_001351030.1	36.2	-	-	No significant similarity found
3.XP_001351047.1	80.25	77.78	72.15	No significant similarity found

No amino acid sequence homology found in other Plasmodium strains

### Analysis of conserved identity with other Plasmodium strains

This step was carried out in order to identify antigens which can provide cross-protection among Plasmodium species. Here, a BLASTp analysis was performed to assess the individual sharing of the selected hypothetical proteins among *Plasmodium vivax, Plasmodium ovale*, and *Plasmodium yoeli.* The alignment showed that protein, XP_001351047.1 from *Plasmodium falciparum* shared significant sequence identity, i.e., 80%, 77.78%, and 72.15% with *Plasmodium vivax, Plasmodium ovale*, and *Plasmodium yoeli*, respectively. XP_001351030.1 protein had 36.2% homology to *Plasmodium vivax* while *Plasmodium falciparum* protein XP_001350955.1 did not show any sequence similarity with other Plasmodium species (Table 2).

### Protein physico-chemical parameter analysis

ProtParam analysis show that the molecular weight, pI, II, EC, and GRAVY of the 3 hypothetical proteins ranged between 9.58 and 27.85 kDa, 5.01 and 6.71, 24.47 and 57.78, 62.59 and 98.21, 5960 and 23380, and − 1.156 to − 0.440, respectively. All the three hypothetical proteins had a half-life of 10 h in bacterial host (*Escherichia coli*). The grand average of hydropathy (GRAVY) value for a peptide or protein is the sum of hydropathy values of all the amino acids, divided by the number of residues in the sequence. A GRAVY value is a good indicator of the hydrophobicity of the protein. The lower GRAVY values of our HPs indicated that they have better interaction with water [49].

### B cell epitope prediction

For this analysis, three algorithms namely BCpreds server (BCpred and amino acid pair prediction methods) and ABCpred were utilized. BCpred algorithms generated 20-mer sequences of B cell epitopes with specificity of 70% whereas ABCpred generated 16mer B cell epitopes at a score of 0.51. The combination of BCpred, ABCpred, and VaxiJen servers allowed the prediction of 21 overlapping antigenic B cell epitopes from three hypothetical proteins. Out of the 21 antigenic B cell epitope, 15 were neither allergenic nor toxic. Antigenic B cell epitopes from the selected hypothetical proteins of *P. falciparum* are presented in Table 4.

### Prediction of T cell epitopes

CTLPred server predicted a total of 19 cytotoxic T cell epitopes from the three HPs studied. A total of 13 out of 19 cytotoxic T cell epitope regions were predicted as antigens by VaxiJen server. Of these 13 antigenic epitopes, 7 were found in XP_001350955.1, 4 and 2 epitopes were in XP_001351030.1 and XP_001351047.1, respectively. Out of the 13 antigenic CTL epitopes, 8 epitopes were immunogenic (Table 5). And of the 8 antigenic and immunogenic CTLs, only 2 epitopes (bolded in Table 5) were neither allergenic nor toxic.

### Prediction of helper T-lymphocyte (HTL) epitopes

The IEDB MHC-II binding tool using NN-align method predicted a total of 61 HTL epitopes from the three hypothetical proteins. Thirty out of 61 were antigenic. Twelve out of 30 antigenic HTL epitopes were predicted to induce at least 2 cytokines (interleukin 4, interleukin 10, and interferon gamma). Five HTL peptides (bolded) from XP_001351030.1 protein are predicted inducers of all the three cytokines while no epitope from XP_001350955.1 and XP_001351047.1 proteins was able to induce at least two cytokines (Table 6).

### Protein–protein function prediction

**Fig. 2:**
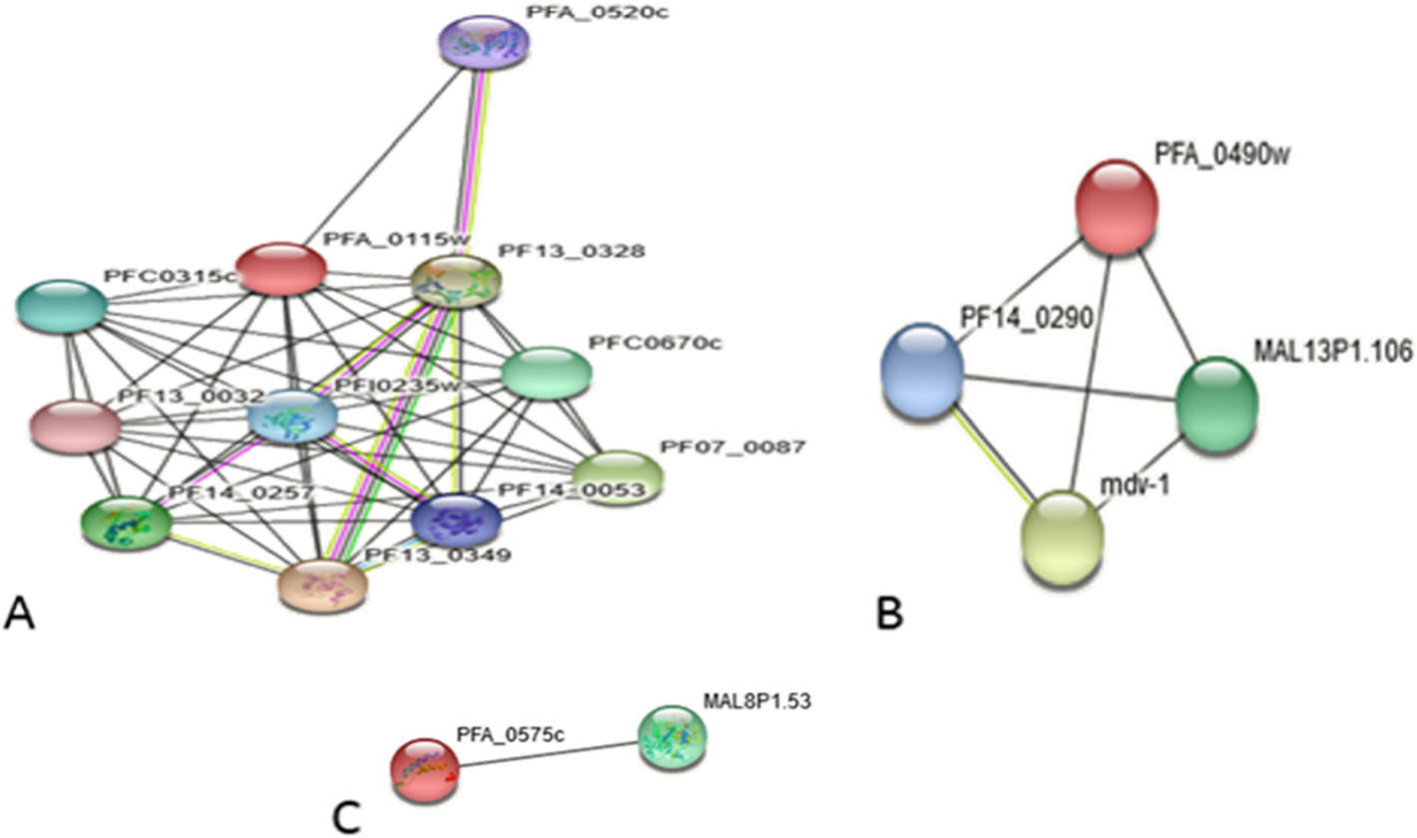
Protein–protein interaction network of three hypothetical proteins of *Plasmodium falciparum* generated by STRING web server. **A** XP_001350955. **B** XP_001351030. **C** XP_001351047.1

Protein–protein interaction networks were analyzed by STRING 10.5 server and revealed 10, 3, and 1 potential interacting protein associates (Fig. 2A–C) for XP_001350955.1, XP_001351030.1, and XP_001351047.1 based on network parameters including text mining, gene fusion, co-occurrence, co-expression, neighborhood, and databases. Because proteins function by interacting with other proteins where they form protein complexes and networks, understanding the complex protein interactions give important clues as to the function of novel proteins. Exploring this type of STRING generated predicted interaction networks can guide future experimental research, e.g., prediction of the possible cellular pathway of the protein of interest. Similar protein–protein interaction study has been previously worked out [50, 51]. XP_001350955.1 closely interacts with nucleoside diphosphate kinase (PF13-0349) at 0.704, XP_001351030.1 interacts with male development protein1 (Mdv-1) at 0.645, and XP_001351047.1 with an uncharacterized protein (MAL8P1.53) at 0.400.

## Discussion

Malaria due to *Plasmodium falciparum* is still a major cause of mortality particularly in the developing countries of Africa and Asia. Until now, research efforts to develop an efficacious malaria vaccine have not yielded. Over the years, there has been rapid development of low-cost sequencing techniques which has led to generation of huge amounts of genomic and proteomic data; however, research on hypothetical proteins (HP) is yet to keep pace with. Currently, over 50% of the *Plasmodium falciparum* proteins have no ascribed function. Characterization of HP may be useful in better understanding the organism’s metabolic pathways, disease progression, drug development, and disease control strategies [52]. With a complete *Plasmodium falciparum* genome sequence [11] and advancement in bioinformatics, it is now possible to identify potential vaccine candidates using reverse vaccinology which reduces the time and cost of designing and identifying vaccine candidates [53]. This study utilized several bioinformatics and immunoinformatic tools for identification and characterization of hypothetical proteins of *P*. *falciparum* for vaccine development. For each protein, different properties and their epitopes were analyzed for possible immune response. For this study, the properties of a good vaccine candidate considered were (1) they should be extracellular surface or cell surface localized to increase their accessibility to immune system surveillance, (2) they must be antigenic, (3) they must not show homology with the human proteins to avoid generation of autoimmune response, (4) they lack or possess one transmembrane (TM) regions to facilitate expression, and (5) they must be non-allergenic. Furthermore, secreted or cell surface antigens are considered good targets for developing vaccine as they are usually antigenic and are responsible for the initial host-pathogen interaction [54]. Secondly, cell surface antigens are easily recognized and do elicit an immune response when used as the target antigens for a vaccine [55] with respect to those pathogens against which a strong B cell response is critical. A signal peptide motif serves to direct the intracellular protein to the extracellular surface of either the plasma membrane and or apical surfaces [56]. Proteomic and immunoinformatic tools revealed hypothetical proteins that could be valuable targets for vaccine development. Based on subcellular localization, antigenicity (VaxiJen score > 0.5), non-relatedness to human proteome (E value = 0.005 and identity at < 35%) and number of transmembrane helices predictions (less than 2), 3 out of 23 hypothetical proteins were identified as potential vaccine candidates against *P*. *falciparum* malaria*.* These three HPs include NCBI accession no. XP_001350955.1, XP_001351030.1, and XP_001351047.1. Subcellular localization of a hypothetical protein is useful to provide insights into their function as different cellular locations represent different functions. The HPs were predicted to be extracellular by WoLF PSORT server, which has a high accuracy in predicting subcellular localization of proteins in eukaryotic organisms [27]. These extracellular proteins can be considered as vaccine targets. However, there is need to update and confirm their exact localization using immunoflourescent assays of electron microscope. VaxiJen server also showed that the selected HPs were immunogenic. The transmembrane localization of the protein positions itself to interact directly with the host’s immune system; therefore, the number of transmembrane domains (TM) is seen as one of the selection criteria for a potential vaccine candidate. However, vaccine targets should possess ≤ 1 TM as it is usually difficult to clone, express, and purify proteins with more than one TM spanning regions. We predicted TM regions using TMHMM and HMMTOP programs and all the three HPs had less than 2 TM regions (Table 1). Since vaccine candidates with similar sequence to the hosts (e.g., human and mouse) may cause autoimmunity [57]. It is therefore imperative that the probable vaccine candidates have no human homologs and hence exclusively present in pathogens and absent in humans. The three selected HPs were submitted to NCBI-BLASTp and all did not show significant similarity with human host (Table 2), suggesting that they could be used for vaccine development without causing autoimmunity. The appropriate physico-chemical properties and stable structure of the potential vaccine candidates are needed to evoke an immune response [58]. The GRAVY value for a peptide or protein is calculated as the sum of hydropathy values of all the amino acids divided by the number of residues in the sequence [38, 59]. All the three hypothetical proteins analyzed had negative GRAVY values (Table 3) clearly indicating their hydrophilic nature and good water solubility property. This information might be useful for localizing these proteins. The molecular weight, isoelectric point, and extinction coefficient of proteins are important in setting-up purification and crystallization experiments [60]. Furthermore, molecular weight is also important in characterizing protein function. Our HPs; XP_001350955.1, XP_001351030.1, and XP_001351047.1 had Mw 13581.48 Da, 27846.86Da, and 9581.76Da respectively. The extinction coefficient of our hypothetical proteins at 280 nm ranges from 5960 to 12,950 M cm with respect to the concentration of cysteine (Cys), Tryptophane (Trp), and Tyrosine (Tyr). The high extinction coefficient of hypothetical proteins is an indicator of presence of high concentration of Cys, Trp, and Tyr. It is defined as a measurement of how strongly a protein absorbs light at a given wavelength. The computed extinction coefficients aid in the quantitative study of protein–protein and protein–ligand interactions in solution [16]. Instability index is a measure of the in vivo stability of a protein and therefore an instability index smaller than 40 is believed to be stable [60, 61]. Two, XP_001350955.1 and XP_001351047.1 of our HPs had an instability index of 38.84 and 24.47 respectively hence are thus likely to be stable, while XP_001351030.1 which had instability index of 57.78 is considered unstable. The aliphatic index is estimated based on the number of aliphatic residues (alanine (Ala), valine (Val), isoleucine (Ile), and leucine (Leu)) in the protein and higher values indicate higher thermo stability over a wide temperature range [62]. Aliphatic index for the hypothetical protein sequences ranged from 62.59 to 98.21. The very high aliphatic index of the protein sequences indicates that these proteins may be stable for a wide temperature range. Thus, all the calculated physicochemical properties could be important for further experimental studies of these HPs. Several reports [63–66] indicate that most of the malaria vaccines work mainly by inducing protective serum antibodies and to some extent CD4+ T cells which is often a sufficient component of vaccine efficacy. Unlike antibodies, however, CD8 T cells alone are also capable of conferring complete sterilizing protection, demonstrating their critical role in pre-erythrocytic immunity [67, 68]. Therefore, both the antigenic B and T cell epitopes are essential for obtaining the maximum immune response through humoral and cell-mediated immunity. The B cell epitopes were identified through ABCpred and Bcpred servers while CTL and HTL cell epitopes were predicted using CTLPred and IEDB-MHC11 web servers respectively and were further validated against antigenic property through VaxiJen server. This is based on the idea that the development of a peptide vaccine largely relays on identifying immunodominant epitopes that can induce specific immune responses without the need of involving whole microorganism. From the three HPs, a number of antigenic B, cytotoxic and helper T cell epitopes were identified which could potentially be used for designing an epitope based vaccine against *P. falciparum* malaria (Tables 4, 5, and 6).

**Table 3:** Physicochemical properties of the hypothetical proteins of *Plasmodium falciparum*

NCBI accession no.	Amino acid	Molecular weight	pI	Instability index (II)	Aliphatic index (AI)	Extinction coefficient (EC)	GRAVY	Half life (h)
XP-001350955.1	117	13581.48	5.01	38.84	98.21	5960	− 0.440	10
XP-001351030.1	234	27846.86	6.55	57.78	70.38	23380	− 0.824	10
XP-001351047.1	81	9581.76	6.71	24.47	62.59	12950	− 1.156	10

*pI* isoelectric point, *GRAVY* grand average of hydropathy

**Table 4:** Final list of potential B-cell epitopes of the three selected hypothetical proteins of *Plasmodium falciparum*

Protein ID	B-cell epitope	Start position	ABCpred	VaxiJen	AllergenFP	ToxinPred
XP_001350955.1	TNIIDESEQENSIDLS	85	0.89	0.5752	Non-allergen	Non-toxin
	TQPISKKTSVEEKLKE	56	0.80	0.7490	Non-allergen	Non-toxin
	SIDLSDYTNIGSSLNP	96	0.77	1.0217	Non-allergen	Non-toxin
	LAQCLNTQPISKKTS	49	0.65	0.5782	Non-allergen	Non-toxin
	FSSIKIFLFFIVLEIL	4	0.52	0.7748	Non-allergen	Non-toxin
XP_001351030.1	YDEEDIDTEKASPLNP	95	0.90	0.5239	Non-allergen	Non-toxin
	RQKNKKKVLHDASLFD	192	0.82	0.6868	Non-allergen	Non-toxin
	PLNPYLAPSFVEMQSK	107	0.72	0.5705	Non-allergen	Non-toxin
	DQHIKKMNNKRICKNG	207	0.69	0.8737	Non-allergen	Non-toxin
	LLIISCALFFLFKRSE	13	0.69	0.6245	Non-allergen	Non-toxin
	DNMNNNDDNNNVKKYK	79	0.64	0.8050	Non-allergen	Non-toxin
XP_001351047.1	QNTIKAHVEANDECKE	10	0.91	1.3329	Non-allergen	Non-toxin
	KKEKYLKCFNNWYKNN	26	0.89	0.5078	Non-allergen	Non-toxin
	TQACDDYYEDYQICVL	48	0.81	0.8927	Non-allergen	Non-toxin
	CFNNWYKNNFLKGDLT	33	0.63	1.2766	Non-allergen	Non-toxin

**Table 5:** List of cytotoxic T cell epitopes predicted from the three hypothetical proteins of *Plasmodium falciparum*

Protein ID	Peptide	Start	Score (ANN/SVM) (0. 51/ 0.36)	Antigenicity score (>0.5)	Immunogenicity score (IEDB>0)	Allergenicity	Toxicity
XP_001350955.1	**RQNIPIRSL**	**42**	**0.88/0.96567173**	**0.7996**	**0.19332**	**No**	**No**
	FIVLEILLL	13	0.87/0.70629735	1.1767	0.20918	Yes	No
	DRNEKQTNI	79	0.91/0.58847506	1.6421	-0.19141	No	No
	FLFFIVLEI	10	0.49/0.92826689	1.5459	0.3734	Yes	No
	CLNTQPISK	53	0.91/0.38273127	0.7369	-0.07062	Yes	No
	AQCLNTQPI	51	0.89/0.35780961	0.9096	-0.10266	Yes	No
	KKTSVEEKL	61	0.80/0.42325026	0.7333	-0.06092	No	No
XP_001351030.1	VESIFYENV	57	0.66/0.96673418	0.8440	0.27146	Yes	No
	**ASLFDQHIK**	**203**	**0.97/0.54674556**	**1.3009**	**0.13182**	**No**	**No**
	ASPLNPYLA	105	0.75/0.69145562	0.9599	-0.0411	No	No
	ISCALFFLF	16	0.82/0.57119502	1.2939	0.21359	Yes	No
XP_001351047.1	YYEDYQICV	54	0.75/0.41137609	1.2069	0.023	Yes	No
	FNNWYKNNF	34	0.58/0.3902168	1.0489	0.00495	Yes	No

**Table 6:** Helper T-lymphocyte epitopes predicted from the three hypothetical proteins of *Plasmodium falciparum*

Protein	HTL peptide	Position	NN-align P. rank	Antigenicity	IL10pred	IL4pred	IFNepitope
XP_001350955.1	LSDYTNIGSSLNPDD	29–43	3.5	1.3058	Non-inducer	Non-inducer	Negative
	SDYTNIGSSLNPDDI	30–44	3.7	1.0481	Non-inducer	Inducer	Negative
	DLSDYTNIGSSLNPD	28–42	4.1	1.2586	Non-inducer	Non-inducer	Negative
XP_001351030.1	**NEAKMDSFIYQIYMM**	**91–105**	**2.8**	**1.3318**	**Inducer**	**Inducer**	**Positive**
	**EAKMDSFIYQIYMMK**	**92–106**	**1.9**	**1.4314**	**Inducer**	**Inducer**	**Positive**
	**AKMDSFIYQIYMMKS**	**93–107**	**1.8**	**1.0428**	**Inducer**	**Inducer**	**Positive**
	**KMDSFIYQIYMMKSE**	**94–108**	**1.7**	**1.0074**	**Inducer**	**Inducer**	**Positive**
	**MDSFIYQIYMMKSEF**	**95–109**	**1.5**	**1.0892**	**Inducer**	**Inducer**	**Positive**
	EYNEAKMDSFIYQIY	89–103	3.4	1.1536	Inducer	Inducer	Negative
	YNEAKMDSFIYQIYM	90–104	3.3	1.3256	Inducer	Inducer	Negative
	DSFIYQIYMMKSEFD	96–110	0.46	0.7763	Non-inducer	Inducer	Positive
	SFIYQIYMMKSEFDK	97–111	0.18	0.6950	Non-inducer	Inducer	Positive
	FIYQIYMMKSEFDKN	98–112	0.18	1.0801	Non-inducer	Inducer	Positive
	IYQIYMMKSEFDKNL	99–113	0.33	0.7683	Non-inducer	Inducer	Positive
	YQIYMMKSEFDKNLN	100–114	0.63	0.9757	Inducer	Inducer	Negative
XP_001351047.1	KYLKCFNNWYKNNFL	29–43	4.9	0.6516	Non-inducer	Inducer	Negative

*IL4* interleukin 4, *IL10* interleukin 10, *IFN* interferon

**Fig. 3:**
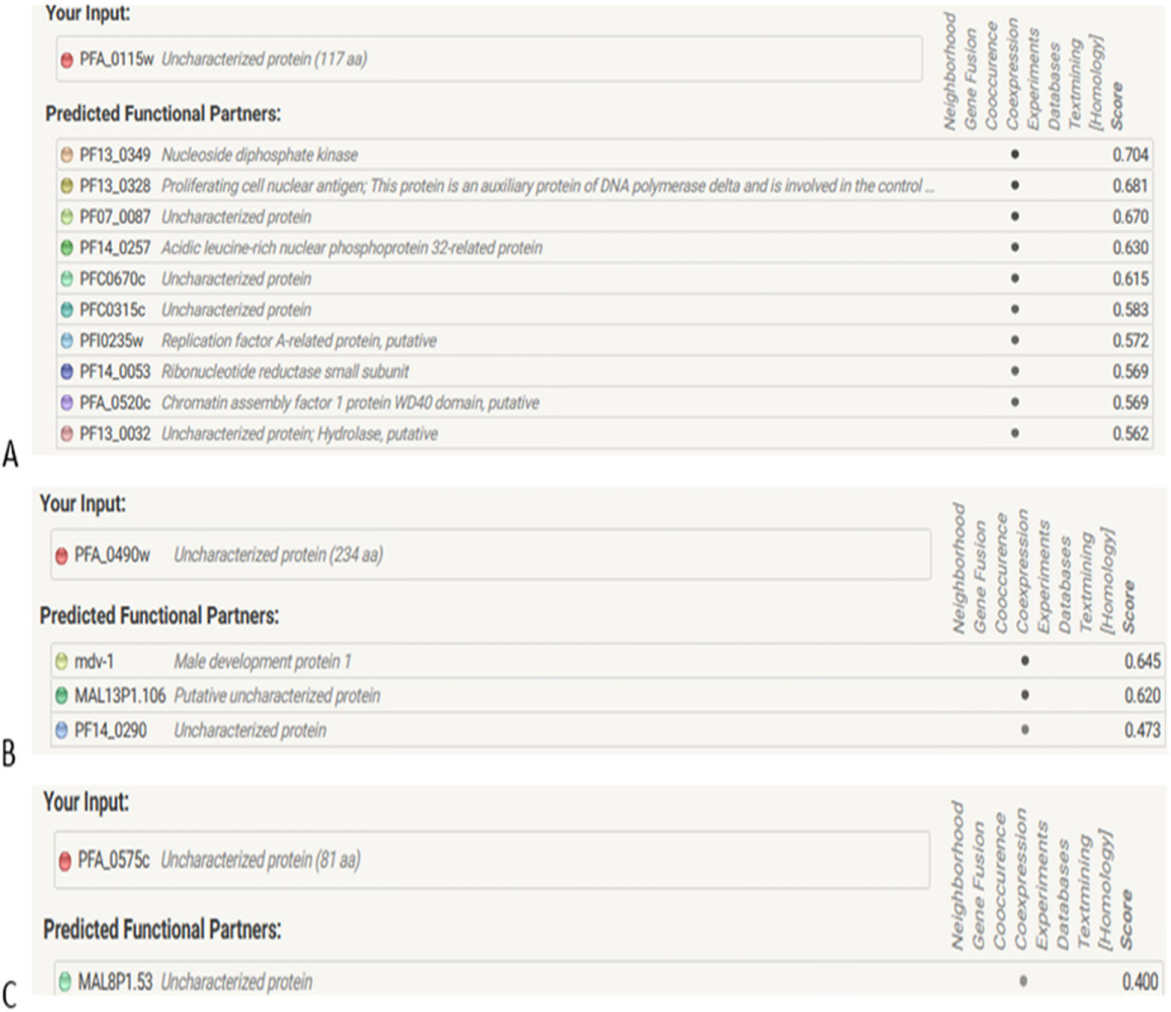
Screenshots from STRING server of predicted functional partners with query sequences. Top interacting partners have been displayed for **A** XP_001350955. **B** XP_001351030. **C** XP_001351047

The characterization of protein–protein interactions provides insights into their biological and cellular functions in the cell. Generally, the function and activity of a protein are often modulated by other proteins with which it interacts. A typical example are the molecular processes of DNA replication, transcription, translation, cell signaling, and cell cycle control among others which are performed by large number of proteins organized by their protein–protein interactions [69]. Currently, protein–protein interaction databases are increasingly becoming important resource for investigating biological networks and pathways in cells. For functional protein–protein networks, STRINGv10.0 was used for the prediction of the interaction between our hypothetical proteins with other partners (Fig. 2). The protein frameworks are derived from various experimental data, analysis of gene, the gene fusion neighborhood, co-occurrence, and co-expression that is curated from various pathway databases [70]. The top partner proteins with an interaction score > 0.4 were applied to construct the PPI networks to query hypothetical proteins. Protein XP_001350955.1 interacts with 10 proteins: nucleoside diphosphate kinase (NDK), proliferating cell nuclear antigens (PCNA), uncharacterized protein (PF07_0087), acidic leucine-rich nuclear phosphoprotein 32-related protein, uncharacterized protein (PFC0670c), uncharacterized protein (PFC0315c), replication factor A-related protein putative, ribonucleotide reductase small subunit, chromatin assembly factor 1 protein WD40 domain putative, and uncharacterized protein; hydrolase putative. Nucleoside diphosphate kinases are enzymes required for the synthesis of nucleoside triphosphates. Proliferating cell nuclear antigen (PCNA) plays an essential role in DNA replication and repair machinery as the processivity factor for DNA polymerase δ and ε [71]. Protein XP_001351030.1 partners with male development protein1, which is important in female gametocyte activation [72]. It also interacts with putative uncharacterized protein (MAL13P1.106) and uncharacterized protein (PF14_0290). Protein XP_001351047.1 is found to interact with only one protein: uncharacterized protein (MAL8P1.53). The predicted functional partner proteins, alongside their confidence scores for each hypothetical protein involved in this study, are summarized in Fig. 3. The protein–protein interactions are critical for almost every process in a living cell; therefore, information generated herein about the interactions of our HPs with other proteins could shed insight into understanding the parasite pathogenesis and can provide the basis for novel vaccine approaches.

However it is essential that the selected vaccine candidates along with their epitopes be further validated for their immunogenicity and protective efficacy experimentally if they are to be used for future vaccine development against *P*. *falciparum* malaria.

## Conclusion

Reverse vaccinology is a promising strategy for the screening and identification of antigenic antigens with potential capacity to elicit cellular and humoral immune responses against *P*. *falciparum* infection. In this study, three hypothetical proteins were selected through computational methods and verified as potential vaccine candidates against *P*. *falciparum* malaria. We therefore recommend further in-depth immunoinformatics and structural biology approaches together with in vitro and in vivo experiments to validate their immunogenicity and protective efficacy to completely decipher the vaccine targets against malaria.

## Data Availability

All the data and material generated and analyzed in this study have been included in this manuscript.

## Abbreviations

RV: Reverse vaccinology
NCBI: National center for information and biotechnology
TM: Transmembrane helices
Ab: Antibody
GRAVY: Grand average of hydropathy
HTL: Helper T cells
AAP: Amino acid pair
IEDB: Immune epitope database
CTL: Cytotoxic T-lymphocytes
MHC: Major histocompatibility complex
BLAST: Basic local alignment search tool
IL: Interleukin
IFN-ɣ: Interferon gamma
ANN: Artificial neural network
SVM: Support vector machine
TMHMM: Trans membrane hidden Markov model
Mw: Molecular weight
PI: Isoelectric point
II: Instability index
EC: Extinction coefficient
HP: Hypothetical proteins
Cys: Cysteine
Try: Tryptophan
Tyr: Tyrosine
Ala: Alanine
Val: Valine
Ile: Isoleucine
NDK: Nucleoside diphosphate kinase
PCNA: Proliferating cell nuclear antigen
